# Microstructure formation and interface characteristics of directionally solidified TiAl-Si alloys in alumina crucibles with a new Y_2_O_3_ skull-aided technology

**DOI:** 10.1038/srep45198

**Published:** 2017-03-23

**Authors:** Jianglei Fan, Jianxiu Liu, Shen Wu, Shuxia Tian, Hongxia Gao, Shengyong Wang, Jingjie Guo, Xiao Wang

**Affiliations:** 1 Institute of Mechanical and Electrical Engineering, Zhengzhou University of Light Industry, Zhengzhou, 450002, P. R. China; 2 School of Material Science and Engineering, Harbin Institute of Technology, Harbin, 150001, P. R. China; 3 School of Energy and Power Engineering, Zhengzhou University of Light Industry, Zhengzhou, 450002, P. R. China

## Abstract

The microstructure evolution and interface characteristics of a directionally solidified Ti-43Al-3Si (at.%) alloy in an alumina (Al_2_O_3_) crucible with new Y_2_O_3_ skull-aided technology were investigated. The Y_2_O_3_-skull that is in contact with the TiAl-melt is relatively stable, which results in a more controlled reaction between the skull and the melt than in the case of an Al_2_O_3_ crucible is used. A thin reaction layer was formed between the mould and the melt through mutual diffusion. The layer thickness increased with increasing reaction time. The thickness of this layer was less than 80 μm for reaction times up to 5800 s. Y_2_O_3_ particles were not found in the specimen because the mould coating was prepared with fine Y_2_O_3_ powder without a binder, which prevented the Y_2_O_3_ particles splitting from the coating as a consequence of thermal physical erosion. The oxygen content of the TiAl-alloy increased with increasing reaction time. The total oxygen content of the solidified specimen was less than that of the specimen solidified in the Al_2_O_3_ crucibles. This new Y_2_O_3_ skull-aided technology is expected to improve the surface quality of TiAl-alloys and reduce the reaction between the crucible/mould and molten TiAl alloys during directional solidification processing with longer contact times.

γ-TiAl-based alloys have numerous attractive properties: they are lightweight, resistant to oxidation and strong at high temperatures^1^,^2^,^3^,^4^,^5^. These properties meet the demands of next-generation aircraft and automotive engines. The main obstacles that limit commercial applications of TiAl alloys are their inherently brittle nature, low fluidity and high processing cost^6^. Nevertheless, the casting process provides a method with advantages for the manufacture of TiAl alloys when compared to forging and rolling^7^. Various techniques have been applied to investigate the solidification behaviours of TiAl alloys, such as the optical floating zone method^8^,^9^, Bridgman method^10^,^11^,^12^,^13^,^14^, water-cooled copper crucible method^15^,^16^,^17^,^18^,^19^, and electromagnetic confinement method^20^,^21^,^22^. Because of the well-known high reactivity of the melt, the selection of the mould material for casting of TiAl alloys is important.

Until now, a reaction between the metal and crucible was inevitable and led to metal contamination^23^,^24^,^25^,^26^. Hence, imparting the inner face of the mould with high chemical stability, mechanical stability, and thermal stability is a key factor for the precision casting of γ-TiAl alloys^27^,^28^. In the case of TiAl alloys, the materials commonly used for crucibles or moulds are oxide ceramics such as Y_2_O_3_^29^,^30^, CaO^31^, Al_2_O_3_^32^,^33^,^34^,^35^,^36^, ZrO_2_^37^,^38^, CaZrO_3_^39^, AlN^40^ or BN^41^. The reaction between the TiAl alloy melt and crucible can be controlled over a relatively short solidification time using these materials^33^,^42^. Nonetheless, with a longer contact time, the surface quality and microstructure of TiAl alloys are still difficult to effectively control. Some methods that do not include a container, such as optical and induction floating zone furnaces^9^,^43^,^44^,^45^ and the electromagnetic confinement method, are also used for fundamental directional solidification studies of TiAl alloys^22^,^46^. From the perspective of cost, using oxide ceramic crucible/mould remains the best choice for preparing castings of TiAl alloys by. Y_2_O_3_ is regarded as the best crucible material; however, Y_2_O_3_ is expensive and exhibits poor thermal shock resistance^47^. Although CaO is more inert than Al_2_O_3_, moisture accumulation hinders its practical applications^31^. Despite being less stable than Y_2_O_3_ and CaO, Al_2_O_3_ is still the best choice for casting TiAl alloys because of its good thermal shock resistance and lower cost^48^. Therefore, Al_2_O_3_ is often used as the crucible materials for the directional solidification of TiAl alloys^32^,^33^,^34^,^36^. Coatings are commonly applied to Al_2_O_3_ crucible in directional solidification experiments involving TiAl alloys^49^. However, the contamination of Y_2_O_3_ particles by atmospheric oxygen during the long solidification process needs to be minimized.

The purpose of the present work is to investigate the microstructure formation and interface characteristics of directionally solidified Ti-43Al-3Si (at.%) alloys with a new Y_2_O_3_ skull-aided technology under different reaction times. These results will contribute to the surface quality control of investment casting and the directional solidification of TiAl-based alloys.

## Results and Discussion

### Microstructure formation

**Figure 1:**
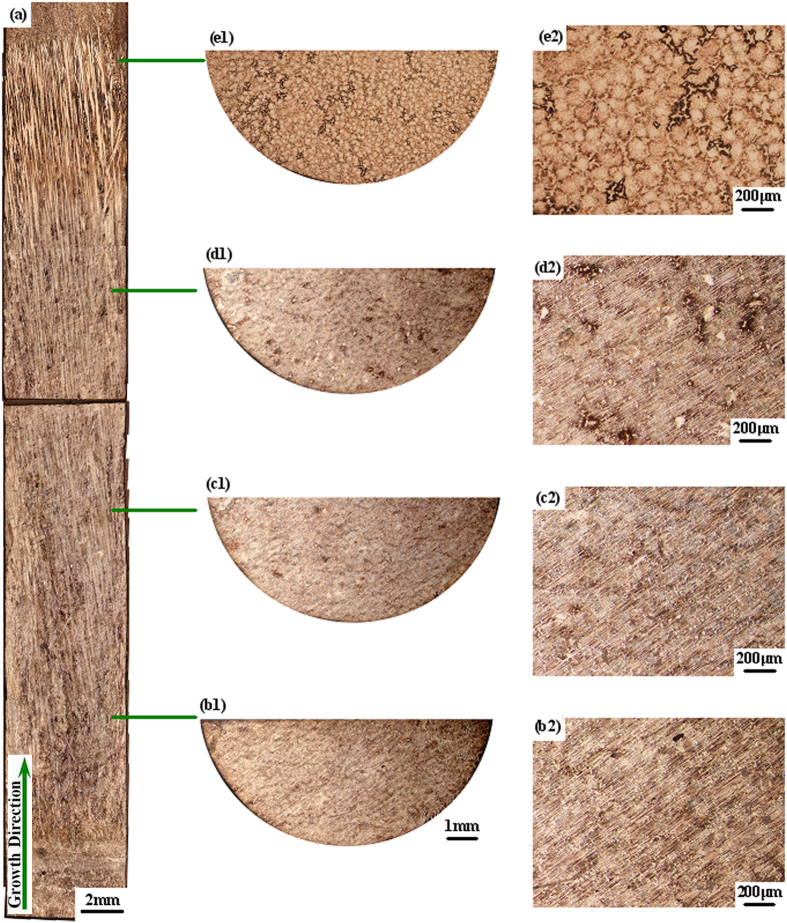
Microstructure of the directionally solidified Ti-43Al-3Si (at.%) at a growth rate of 10 μm/s using Y_2_O_3_ skull-aided technology: (**a**) longitudinal section, (**b**) to (**e**) transverse sections at different distances from the bottom of the specimen at (**b**) 10 mm, at (**c**) 20 mm, at (**d**) 30 mm and at (**e**) 40 mm from the bottom of the specimen. The right sides are high magnification images corresponding to transverse sections at different positions.

The structure of the directionally solidified Ti-43Al-3Si (at.%) alloy is shown in Fig. 1. The macrostructure of the specimen is shown in Fig. 1a. The specimen grew with columnar grains aligned along the drawing direction. The morphology of the solid-liquid interface was preserved by quenching and was cellular under the present solidification conditions. The macrostructures on the transverse sections are shown in Fig. 1b1–e1. These images indicate that the microstructure was uniform throughout the entire specimen. The microstructure of the Ti-43Al-3Si (at.%) alloy after directional solidification is a full-lamellar structure with silicide, as shown in Fig. 1b2–e2.

**Figure 2:**
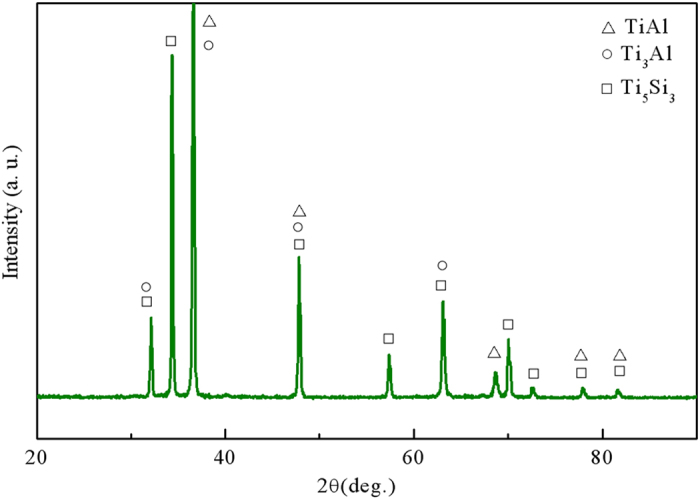
X-ray diffraction pattern of a directionally solidified Ti-43Al-3Si (at.%) alloy, revealing the existence of γ-TiAl, α_2_-Ti_3_Al and ξ-Ti_5_Si_3_ phases.

**Figure 3:**
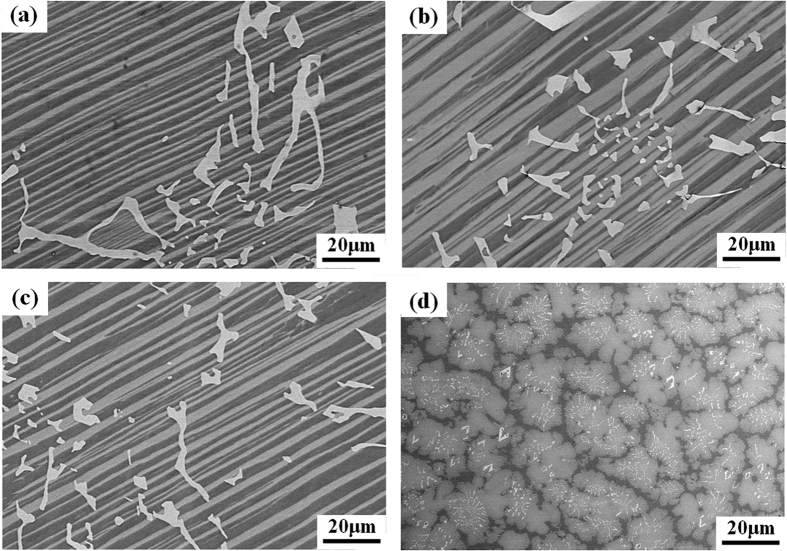
SEM-BSE images on transverse sections of the directionally solidified Ti-43Al-3Si (at.%) alloy with different reaction times (**a**) 2800 s, (**b**) 3800 s, (**c**) 4800 s and (**d**) 5800 s.

**Figure 4:**
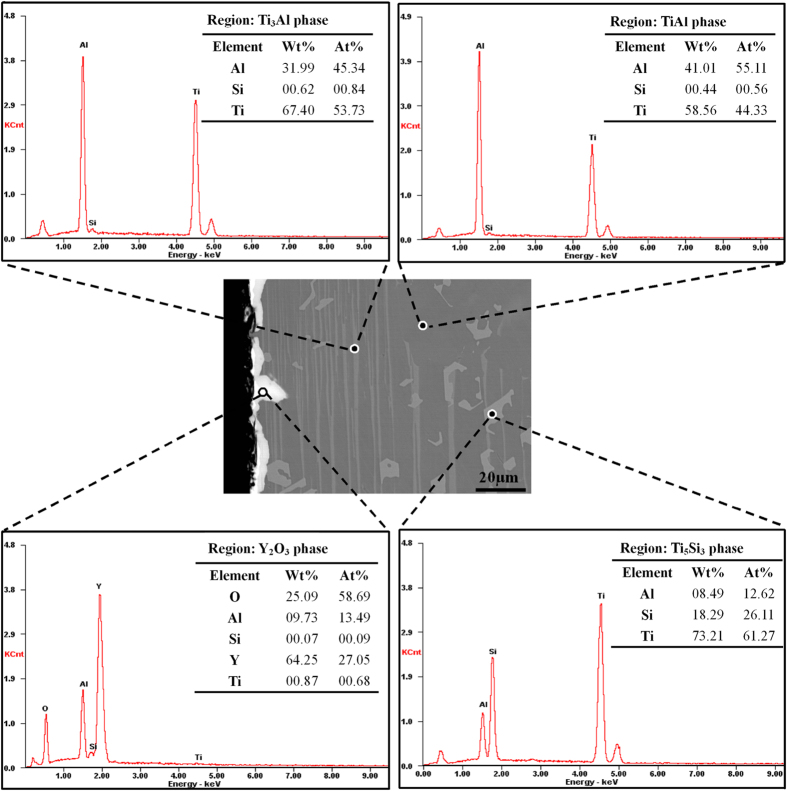
EDX spectrum of phases present near the surface of the Ti-43Al-3Si (at.%) specimen directionally solidified in a Y_2_O_3_-coated Al_2_O_3_ crucible.

According to the XRD pattern shown in Fig. 2, the specimen consisted of TiAl, Ti_3_Al and Ti_5_Si_3_ phases. Y_2_O_3_ particles were not found in the specimen, unlike the results of previous studies^30^. This lack of Y_2_O_3_ particles is attributed to the coating of the mould being prepared using fine Y_2_O_3_ powder without a binder, resulting in fewer Y_2_O_3_ particles being split from the coating via thermal physical erosion. These observations indicate that the poor thermal shock resistance of the Y_2_O_3_ crucible could be improved using this method. Backscattered electron (BSE) mode scanning electron microscopy (SEM) images of the microstructures are shown in Fig. 3. A Composition analysis of the phases was conducted using energy-dispersive X-ray spectroscopy (EDX); the results are shown in Fig. 4. On the basis of the EDX results, the black, grey and white phases were identified as TiAl, Ti_3_Al and Ti_5_Si_3_ phases, respectively.

### Interface characteristics

**Figure 5:**
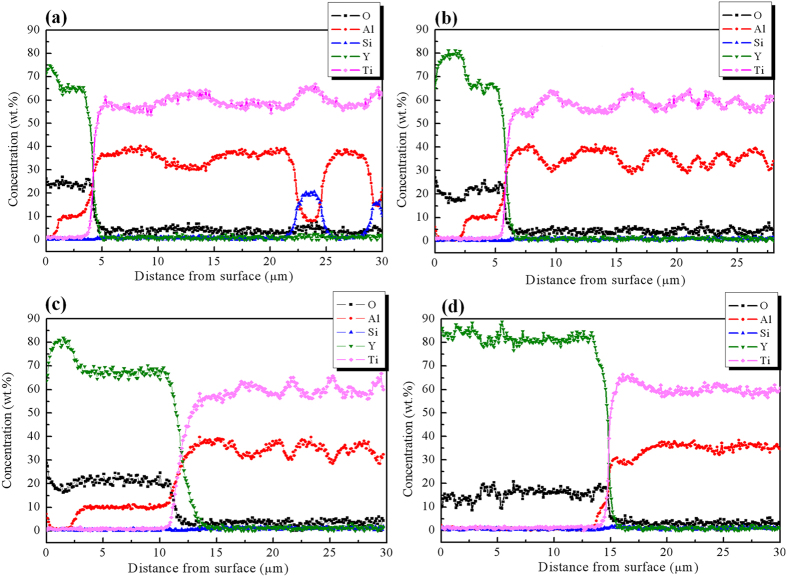
Elemental distribution within the depth from the surface of the directionally solidified Ti-43Al-3Si (at.%) alloys with the following reaction times: (**a**) 2800 s, (**b**) 3800 s, (**c**) 4800 s and (**d**) 5800 s.

The surface morphology of the solidified specimen is shown in Fig. 4. A reaction region with inclusions was not formed as described by Cui^50^. Instead, a discontinuous bright layer with greater compactness on the surface was observed. As shown in Fig. 4, the bright layer consisted of Y, O, and areas of Al. Because the surface of the specimen was cleaned by ultrasonication before observation, the bright layer was a reaction layer between the Y_2_O_3_ skull and the TiAl melt rather than an adhesive layer of Y_2_O_3_ on the surface. The thickness of the reaction layer between the crucible and the melt increased with increasing reaction time, as shown in Fig. 5. The maximum thickness of the reaction layer was less than 15 μm, as shown in Fig. 5d. The severe reaction between the Al_2_O_3_ crucible and the melt for the directionally solidified specimen did not occur with a long reaction time (5800 s). This may be due to the high stability of Y_2_O_3_ in the TiAl melts diminishing the reaction between the mould and the TiAl melt.

Variations in composition from the surface to the interior of the solidified specimen as a function of reaction times are shown in Fig. 5. The oxygen and yttrium contents were greater on the surface than inside the specimen. The oxygen and yttrium contents decreased rapidly from the surface towards the interior of the specimen. Hence, a reaction layer existed between the mould and the melt. According to Fig. 5, an Al-segregation region was not formed at the surface of the specimen, which is different from the results reported by Cui^50^. This difference arises from the specimen being melted and directionally solidified in the Al_2_O_3_ crucible with the Y_2_O_3_ skull under controlled solidification parameters, specifically, the temperature gradient and the growth rate. The cooling rate at the surface of the specimen was lower, and the composition did not affect the solidification path.

The reactivity of the mould and the TiAl melt is related to the Gibbs free energy changes during the reaction. Kostov^51^ recently reported that the standard Gibbs free energy of Y_2_O_3_ is more negative than that of Ti oxides (i. e., TiO, Ti_2_O_3_, TiO_2_). Thus, the molten TiAl alloy does not react with the Y_2_O_3_ skull spontaneously. However, a reaction layer is formed in the directionally solidified specimen. Saha *et al*.^52^,^53^ have indicated that the standard Gibbs free energy could not fully explain the reactivity of the oxides and the molten metal. According to previous research results^30^,^50^ the increased oxygen in the specimen during solidification is dependent on the dissolution of the Y_2_O_3_ according to the following reaction:


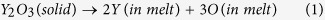


On the basis of the data presented in Fig. 5, the thickness of the surface layer increased with increasing growth distance or, more specifically, with increasing reaction time between the Y_2_O_3_ layer and the melt. Kuang^47^ reported that the relationship between the width of the reaction layer and the reaction time can be represented as follows:


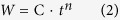


where *n* is the time exponent and *C* is a constant for the given alloy and mould material. For the Ti-48Al-2Cr-2Mn (at.%) alloy, the values of *n* and *C* were 0.52 and 1.74, respectively^47^. The thickness of the reaction layer increased exponentially, as a function of equation (2), with increasing reaction time.

**Figure 6:**
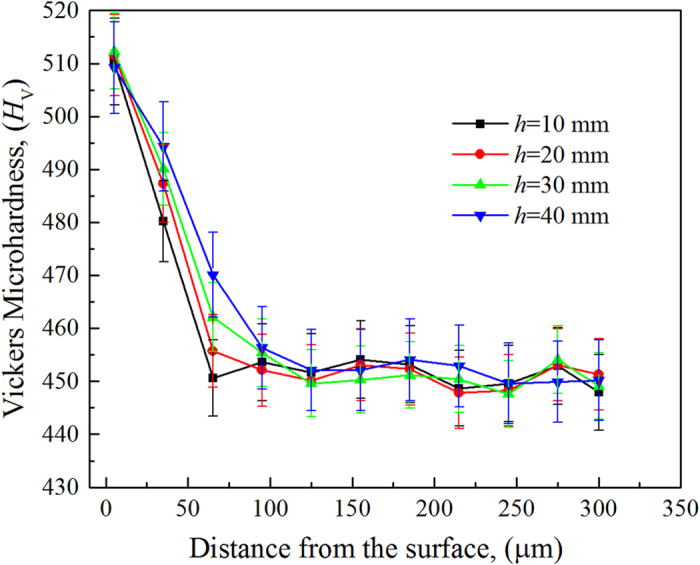
Microhardness profiles of the hardened layer at the surface of the Ti-43Al-3Si (at.%) alloy directionally solidified in an alumina crucible with Y_2_O_3_ skull-aided technology, as measured at different distances (*h*) from the bottom of the specimen.

To evaluate the changes in the mechanical properties from the surface to the inner core of the directionally solidified specimen, we measured the specimen’s microhardness. The microhardness variation from the surface to the centre of the specimen is shown in Fig. 6. A hardened layer at the surface with a hardness of 480–510*H*_V_ was observed, which is greater than that of the inner core of the specimen (approximately 450*H*_V_). According to the observation of the surface microstructure (Fig. 4), Y_2_O_3_ particles did not appeared in the specimen because the physical erosion of the Y_2_O_3_ skull was alleviated or eliminated. The greater hardness of the surface may be attributed to the solid-solute phase of oxygen that originated from the dissolution of the Y_2_O_3_ skull, as described by equation (1). The layer thickness increased with increasing reaction time. The thickness of this layer was less than 80 μm for reaction times up to 5800 s (46.7 minutes). Hence, we determined the surface contamination layer was less than 80 μm thick. This Y_2_O_3_ skull-aided technology is useful for the surface quality control while the casting of TiAl alloys with longer reaction times.

### Variation of the oxygen content during solidification

**Figure 7:**
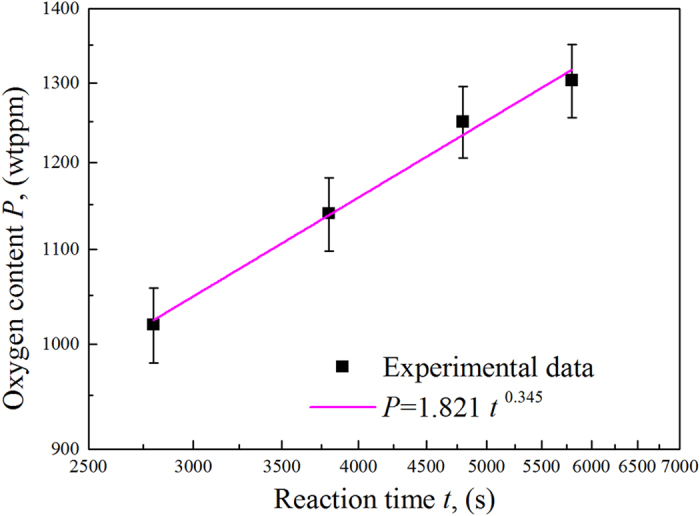
Variation in the oxygen content in the bulk alloy versus the reaction time for Ti-43Al-3Si (at.%) directionally solidified in an alumina crucible with Y_2_O_3_ skull-aided technology.

The total oxygen content of the directionally solidified Ti-43Al-3Si (at.%) alloys increased with increasing reaction time, as shown in Fig. 7. The oxygen content of the specimen directionally solidified in the Y_2_O_3_ skull-aided crucible (approximately 1019–1303 ppm) was lower than that of the specimen directionally solidified in an Al_2_O_3_ crucible studied by Fan (approximately 2600 ppm)^49^.

According to the model established by Lapin^30^, the variation in the oxygen content with reaction time in the directionally solidified TiAl alloy can be expressed as:


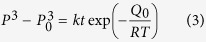


where *P* is the oxygen content in the specimen after directional solidification, *P*_0_ is the original oxygen content of the specimen, *k* is a constant, *t* is the reaction time, *Q*_0_ is the activation energy, *R* is the universal gas constant, and *T* is the temperature of the melt.

In the current experiment, the value of *P*_0_ (419 ppm) was smaller than *P* (>1000 ppm). More specifically, the value of 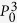 ≪ *P*^3^. If the value of *P*_0_ is ignored, the value of *P* can be simplified as


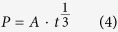


where 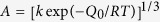.

By regression analysis, the relationship between the oxygen content and the reaction time is obtained as


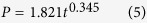


The regression coefficient was *R*^2^ = 0.9901. The value of *t* = 0.345 agrees well with the value of *t* = 1/3 in Lapin’s model^30^.

## Conclusions

1. A new Y_2_O_3_ skull-aided technology was developed to reduce the reaction between the crucible and TiAl alloy melt during the directional solidification process with longer reaction times.
2. After directional solidification, the specimen consisted of TiAl, Ti_3_Al and Ti_5_Si_3_ phases. Y_2_O_3_ particles were not found in the specimen because the Y_2_O_3_ particles that split from the coating due to thermal physical erosion were markedly reduced.
3. The oxygen content *P* increased with increasing reaction time according to the equation *P* = 1.821*t*^0.345^.
4. The hardened layer at the surface was less than 80 μm thick, which is acceptable for the surface quality control of near-net shapes and investment casting of TiAl-based alloys.

## Methods

The master ingot with a nominal composition of Ti-43Al-3Si (at.%) was prepared using cold crucible induction technology in an argon environment; Ti (99.7%), Al (99.9%) and Si (99.9%) were used as the raw materials. The actual chemical composition was obtained by X-ray fluorescence analysis (Axios PW 4400). The oxygen content of the alloy ingot was measured using a CHNS/O elemental analyser. The analysed samples were approximately ∅5 mm × 10 mm. The oxygen content was obtained by measuring the CO_2_ present via using infrared detection. Table 1 shows the measured composition of the prepared alloy. The samples used for the directional solidification experiments were rods (∅6 mm × 100 mm) cut from the ingot using a wire electrical discharge machine.

**Figure 8:**
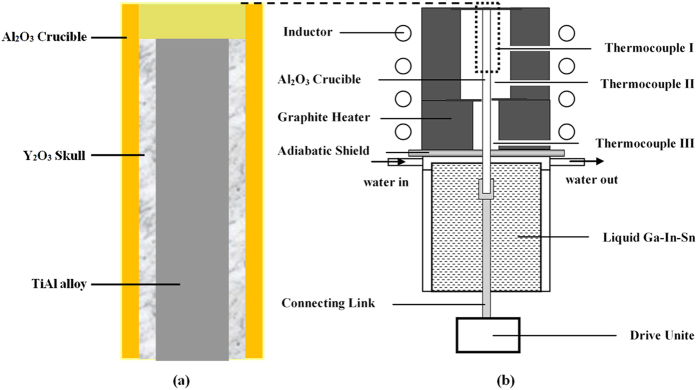
Schematics of (**a**) the specimen with skull-aided technology and (**b**) the Bridgman-type directional solidification furnace.

To minimize the reaction between the Al_2_O_3_ crucible and the melt, a skull-aided technology was used for the directional solidification of the TiAl-based alloy. This technology used fine mixed powders (nanoscale and microscale) of high-purity Y_2_O_3_ without a binder to fill in the interspaces between the alumina crucible and the specimen under a certain pressure. A schematic of the technology is shown in Fig. 8a. The pressure and density of the Y_2_O_3_ skull were carefully controlled. After the specimen and crucible were heated, the Y_2_O_3_ coat became an integral skull and separated the melt from the Al_2_O_3_ crucible.

The directional solidification experiments were performed in a Bridgman-type system, as shown in Fig. 8b. The samples were placed into 99.99% pure alumina crucibles with a 6.5/7.5 mm diameter (inside/outside diameter) Y_2_O_3_ skull and a length of 100 mm. Because of the reactivity of the molten TiAl alloy, the temperature gradient was measured by W/Re thermocouples that were positioned near the outside surface of the alumina tubes to avoid damaging the thermocouple, as shown in Fig. 8b. The chamber of the directional solidification furnace was evacuated to 10^−4^ Pa and filled with argon to avoid oxidation. The specimen was heated to 1823 K over 2 h and thermally stabilized for 1800 s. The specimen was then directionally solidified with a growth rate of 10 μm/s. The growth length of the samples was 40 mm during the directional solidification process. At the end of the experiment, the sample was quenched with a liquid Ga-In-Sn alloy to restore the solid/liquid interface.

The reaction time *t*_2_ during directional solidification was calculated by *d/V*, where *d* is the growth distance and *V* is growth rate. Because the time needed to thermally stabilized the specimen was *t*_1_ = 1800 s, the total reaction time *t* was *t* = *t*_1_ + *t*_2_.

The specimens were cut, polished and etched with a solution of 10 mL HF, 10 mL of HNO_3_, and 180 mL of H_2_O for further analysis. Both optical microscopy (OM) and SEM with the BSE mode were used to characterize the microstructure of the specimens. The phases were identified using a Rigaku D/max-RB X-ray diffractometer equipped with a monochromatic Cu-Kα radiation source. The elemental distributions were analysed by electron probe microanalysis (EPMA) under an acceleration voltage of 15 kV (±2% to 5%).

To evaluate the thickness of the hardened layer, the microhardness values were measured on transverse sections of the specimens with a test load of 100 g, and a dwell time of 10 s. The adopted values of the microhardness were the average values of at least 10 tests on each sample.

## Additional Information

**How to cite this article:** Fan, J. *et al*. Microstructure formation and interface characteristics of directionally solidified TiAl-Si alloys in alumina crucibles with a new Y_2_O_3_ skull-aided technology. *Sci. Rep.* **7**, 45198; doi: 10.1038/srep45198 (2017).

**Publisher's note:** Springer Nature remains neutral with regard to jurisdictional claims in published maps and institutional affiliations.

## Figures and Tables

**Table 1:** Chemical composition of the Ti-43Al-3Si (at.%) ingot.

Element	Ti	Al	Si	O
at.%	Balance	43.27	3.08	0.10 (419 ppm)
